# The Role of Mitotic Slippage in Creating a “Female Pregnancy-like System” in a Single Polyploid Giant Cancer Cell

**DOI:** 10.3390/ijms24043237

**Published:** 2023-02-06

**Authors:** Kristine Salmina, Ninel Miriam Vainshelbaum, Madara Kreishmane, Inna Inashkina, Mark Steven Cragg, Dace Pjanova, Jekaterina Erenpreisa

**Affiliations:** 1 Cancer Research Division, Latvian Biomedical Research and Study Centre, LV-1067 Riga, Latvia; 2 Faculty of Biology, The University of Latvia, LV-1586 Riga, Latvia; 3 Centre for Cancer Immunology, Faculty of Medicine, University of Southampton, Southampton SO16 6YD, UK

**Keywords:** cancer, polyploid giant cell, resistance to treatment, mitotic slippage, soma-germ transition, maternal germ cell, innate immune response, placental developmental genes, parthenogenesis, budding, female pregnancy system

## Abstract

In our recent work, we observed that triple-negative breast cancer MDA-MB-231 cells respond to doxorubicin (DOX) via “mitotic slippage” (MS), discarding cytosolic damaged DNA during the process that provides their resistance to this genotoxic treatment. We also noted two populations of polyploid giant cells: those budding surviving offspring, versus those reaching huge ploidy by repeated MS and persisting for several weeks. Their separate roles in the recovery from treatment remained unclear. The current study was devoted to characterising the origin and relationship of these two sub-populations in the context of MS. MS was hallmarked by the emergence of nuclear YAP1/OCT4A/MOS/EMI2-positivity featuring a soma-germ transition to the meiotic-metaphase-arrested “maternal germ cell”. In silico, the link between modules identified in the inflammatory innate immune response to cytosolic DNA and the reproductive module of female pregnancy (upregulating placenta developmental genes) was observed in polyploid giant cells. Asymmetry of the two subnuclei types, one repairing DNA and releasing buds enriched by CDC42/ACTIN/TUBULIN and the other persisting and degrading DNA in a polyploid giant cell, was revealed. We propose that when arrested in MS, a “maternal cancer germ cell” may be parthenogenetically stimulated by the placental proto-oncogene parathyroid-hormone-like-hormone, increasing calcium, thus creating a ”female pregnancy-like” system within a single polyploid giant cancer cell.

## 1. Introduction

After Cancer-Testis-Antigens (CTA) were discovered in ovaries and placenta and the link between cancer and gametogenesis became apparent [1,2] Lloyd J. Old published an Editorial entitled “Cancer Is a Somatic Cell Pregnancy” [3]. Recently, we reported mitotic slippage (MS) as a means of discarding cytosolic damaged DNA and a key process for cancer cells undergoing recovery from genotoxic treatment by reversible senescence, polyploidy, and alternative telomere repair [4]. The experiments were carried out in vitro on the triple-negative breast cancer cell-line MDA-MB-231 in response to doxorubicin (DOX). Among other observations, we noted super-giant cells, which reach huge ploidy and display an amoeboid phenotype. They diverge in DNA content from the smaller, de-polyploidising recovery fraction and persist for a few weeks. Their role in the recovery process remained unclear. Therefore, here we devoted a deeper analysis to the origin and function of these two populations in reference to MS over the recovery time-course post-DOX treatment, combining immunofluorescence with bioinformatic methods. The link between the transcriptional modules sensing inflammatory innate immune responses to cytosolic DNA, including the response to viral and bacterial infection and host defence, with the emergence of reproductive modules of maternal soma-to-germ transition, including the maternal placenta development pathways in super-giant cancer cells, was observed bioinformatically and confirmed by immunofluorescence analysis. Thus, we revealed a system of somatic cell pregnancy for cancer as postulated by L.J. Old [3] within a single multinucleated polyploidy giant cell during the response to genotoxic drugs. The data are discussed in view of recent discoveries regarding the role of endogenous retrovirus (HERV) domestication in human evolutionary placental development [5], HERV activation in stressed cancer cells [6], participation in cancer metastases [7,8], and the well-known poor prognosis implications of placental markers in many human cancers. Finally, the implication of these findings for cancer treatment is briefly discussed.

## 2. Results

### 2.1. DNA Cytometry to Identify Appropriate Time Points for Studying the Origin and Features of Small Versus Super-Giant Polyploid Cells by Bulk Transcriptomic Analysis

**Figure 1:**
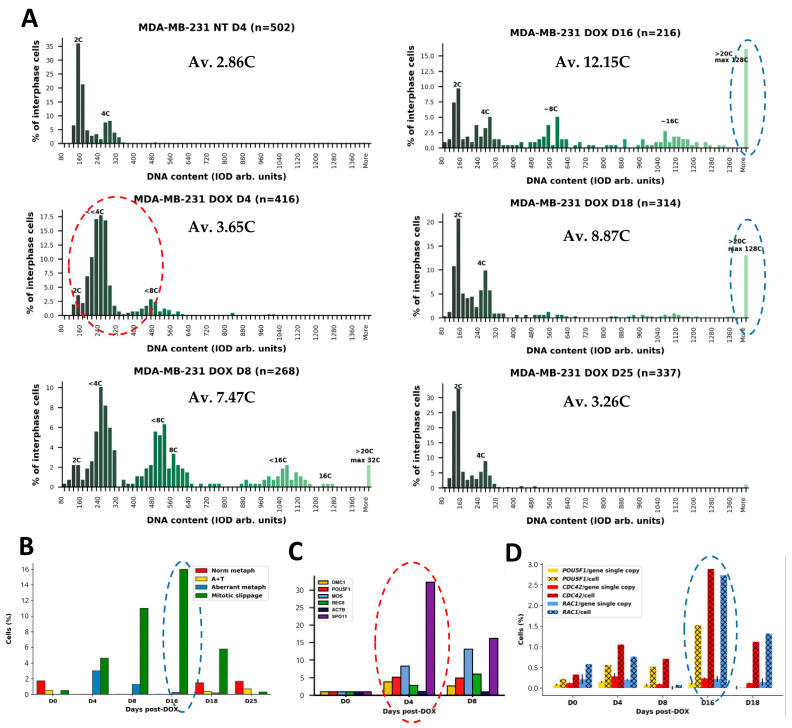
Quantified parameters of MDA-MB-231 cells following DOX treatment: (**A**) Cell cycle changes monitored with in situ DNA cytometry after DOX treatment in a representative experiment; the average ploidy content per cell (AV) is shown for each day. (**B**) Representative differential mitotic counts showing the absence of cell division at the period of increasing MS (D4-D16). (**C**) RT-qPCR results of meiotic gene transcription after DOX treatment, shown as fold change. Representative charts of two independent experiments, with three technical replicates. (**D**) Results of gene transcription evaluation obtained by Selfie digital PCR for three gene transcripts quantified per gene copy and per cell (as transcripts per gene copy multiplied by the average ploidy in the same experiment)—the average of three technical replicates with SEM. Red-dashed circle (in **A**) highlights the period of S-G2-M-delay with DNA under-replication in the late S-phase starting the MS, polyploidisation, and the expression of meiotic genes (in **C**); blue-dashed circles outline the diverged subpopulation of super-giant cells (in **A**) with the highest proportion of MS (in **B**) and the highest proportion of the *POU5F1*, *CDC42*, *RAC1* gene expression per cell (in **D**) (republished from [4] with an open access CC BY 4.0 license).

Based upon DNA cytometry and mitotic counts, we selected time points where we could collect predominantly small and predominantly giant and super-giant polyploid cells. With this approach, in the response of MDA-MB-231 cells to DOX (100 µM, 24 h) treatment, we selected four time points (Figure 1A): (1) non-treated (NT) cells showing a normal cell cycle; (2) the period of replicative stress, S-G2-delay, and DNA under-replication (days 4–5) where MS begins (red-dash circled on Figure 1A; resulting in the first double peak, <8C and 8C; the cycles of MS, increasing ploidy, were further repeating as seen on day 8, resulting in an additional double peak: <16C and 16C); (3) the point of dichotomy between cells starting depolyploidisation (from ~8C to 16C, illustrated by corresponding reductive divisions, shown and discussed below) of the reproductive fraction to the re-establishment of the normal cell cycle (days 16–18), contrasted with the simultaneous accumulation of giant and even super-giant cells (>20C–396C, the highest found ploidy) which continued undergoing MS and increased in number. This second fraction of super-giants is identified with a blued-dashed narrow circle in Figure 1A, while in Figure 1B a similar circle outlines the highest proportion of MS (~16% of cells) counted at this time point. Both fractions gave a high average polyploidy on day 16 (12.15C). Finally (4) the time period of full (or close to full) return to the initial point, with the gradual disappearance of both giant cells and MS (usually seen between days 22 and 29) and represented on Figure 1A by day 25 coinciding with the restart of normal mitoses (Figure 1B) and clonal cell re-growth [4]. The average ploidy content per cell at this time is returning to the control. Up until this recovery, most treated cells expressed the hallmarks of cellular senescence, Sa-β-gal, and IL-6 staining [4].

### 2.2. Differential Gene Expression

**Figure 2:**
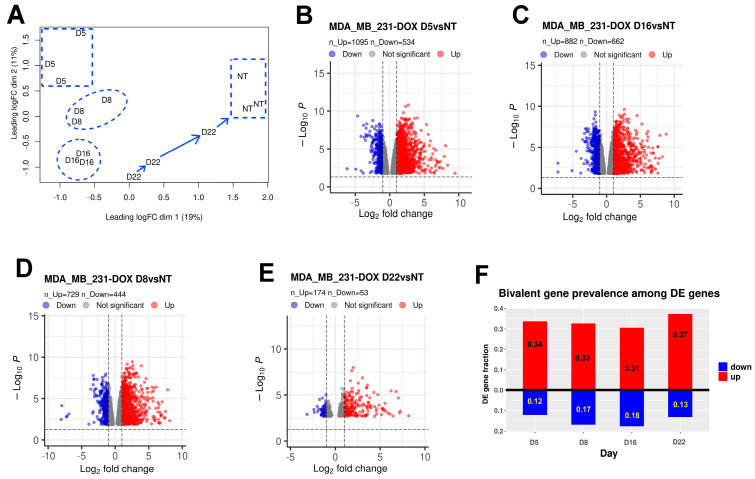
Transcriptome characteristics of DOX-treated MDA-MB-231 compared to non-treated samples from three independent experiments. (**A**) The multidimensional scaling (MDS) plot of MDA-MB-231 RNA-seq samples at days 0 (non-treated, NT), 5, 8, 16, and 22 post-DOX treatment showing (by dashed outline) the separation of the sampled cohorts, with the cohorts on day 22 returning to the NT pattern. (**B**–**E**) Volcano plots of differentially expressed (DE) genes (FDR < 0.05, |LogFC| > 1) on: (**B**) day 5 versus NT; (**C**) day 8 versus NT; (**D**) day 16 versus NT; (**E**) day 22 versus NT. (**F**) The proportions of bivalent genes amongst DE genes, up-and downregulated, respectively, on days 5, 8, 16, and 22 post-DOX.

The full list of differentially expressed (DE) genes after DOX is presented in File S1. In line with the chosen transcriptome sampling time points in three independent experiments, the DE genes formed clusters when presented on the multidimensional scaling plot: for day 5 this was sharply different from the cluster of non-treated (NT) samples and from day 16; for day 16 all three samples were closely converged, different from the NT samples and from day 5; for day 8 the cluster was between those of day 5 and day 16; and for day 22 the samples showed the tendency to return (likely with somewhat different dynamics in each separate case) to the initial NT pattern (Figure 2A). The amount of differentially expressed genes (compared with the NT control) as presented in Figure 2B–D was the highest for days 5 and 16 (similar for day 8), and very much reduced on day 22. The high convergence of the DE pattern for three samples on day 16 was apparently due to the dominant presence of the hyperploid cells, which thus unified the data. It is clear that on days 4–5, along with MS, the cells underwent a very strong change of cell fate. Therefore we first directed our transcriptome analysis to the study of bivalent genes, whose activation is usually characteristic for the developmental changes particularly associated with polyploidy [9].

#### 2.2.1. Quantitation of Up- and Downregulated Bivalent Genes

The activation of bivalent genes (harbouring both transcription-suppressing H3K27me3 and activating H3K4me3 histone H3 modalities at their promoters) is capable of immediately changing their transcription and thus, can impact cell fate. Our results here revealed the enrichment of DOX-treated samples with upregulated bivalent genes. Among the DE genes (presented as volcano plots in Figure 2), the proportion of bivalent genes, as seen in Figure 2F, is >30% among upregulated genes for all time points, and up to 18% among downregulated genes. The revealed up- and downregulated bivalent gene lists are presented in File S2. The proportion of downregulated bivalent genes decreases along with an eight-fold decrease of DE genes on day 22, during recovery. The annotation of upregulated bivalent genes revealed two main module groups—innate immune response to soluble DNA and reproduction—on days 5 and 16 after DOX treatment. 

#### 2.2.2. Gene Ontology (GO) Enrichment Analysis of DE Bivalent Genes Reveals Modules of Cytokine Signaling and Response to Cytosolic DNA

**Figure 3:**
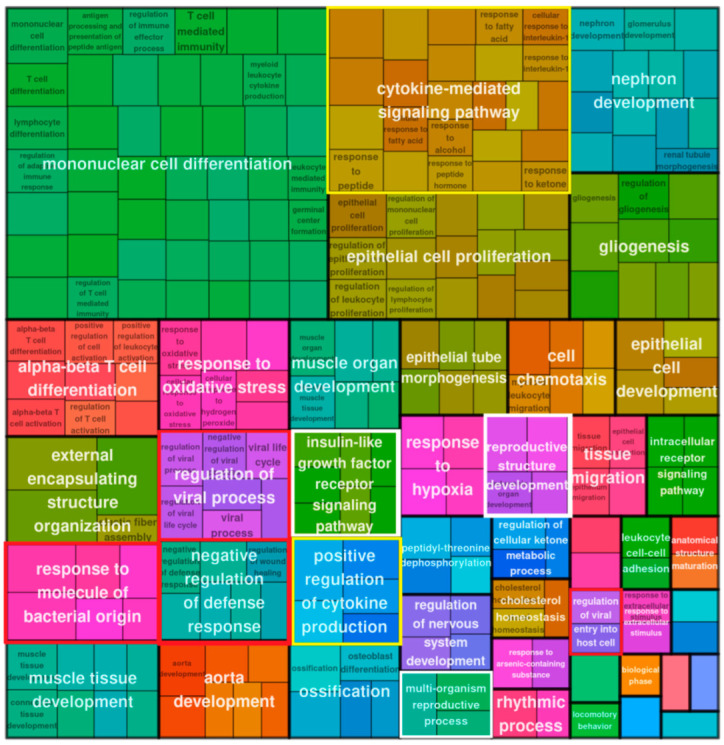
Treemap plot representation of the Gene Ontology Biological Process (GO BP) enrichment analysis (GO modules with hypergeometric test pAdj < 0.05 classed as enriched) for bivalent genes upregulated in MDA-MB-231 cells on day 5 post-DOX.

A GO enrichment analysis of the upregulated bivalent genes of day 5, presented in Figure 3 as a treemap plot, revealed the activation of a stress response and oscillating processes—both typical for reversible senescence [10,11]. 

**Figure 4:**
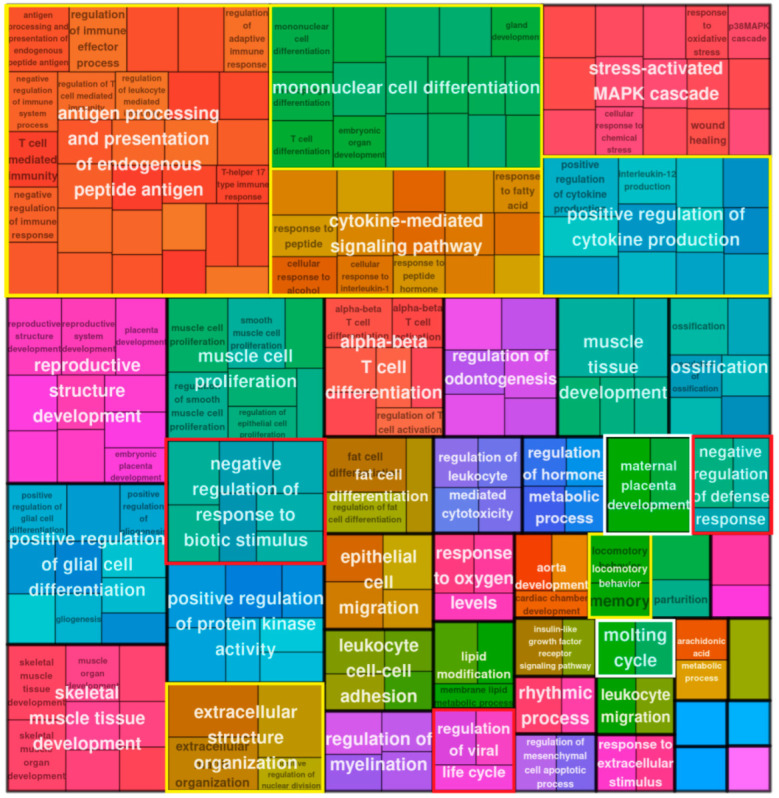
Treemap plot representation of the GO BP enrichment analysis results (GO modules with hypergeometric test pAdj < 0.05 classed as enriched) for bivalent genes upregulated in MDA-MB-231 cells on day 16 post-DOX.

Both settings on day 5 and day 16 revealed several modules describing morphogenesis and a response to the decreased oxygen level. The prominent GO terms of cytokine signaling, regulation of viral process, and response to molecules of bacterial origin (framed by yellow and red boxes) included sensing of soluble DNA by cGAS-Sting or other pathways of adaptive and inflammatory immune response [12]. It was also similar on day 5 and day 16 (Figure 4). 

However, a negative regulation of the defense response and a negative regulation of the biotic stimuli were also found. To better dissect the reaction to cytosolic DNA accompanying MS, we analysed the other sensing pathway triggered by intracellular DNA, the AIM2 inflammasome which may reduce the activation of the STINg pathway [12]. The cytosolic DNA-sensing pathway M39837 was obtained from MSigDB [13] and the list of DE genes for each time point was filtered against it. The M39837 pathway genes present among DE genes are listed in Table 1.

It appears that the AIM2-receptor pathway recognizing cytosolic DNA was activated on day 5 but no further. In contrast, other cytosolic DNA sensors, the receptors of POL II and POL III, were downregulated on days 5–16. I.e., in spite of intense pro-inflammatory signalling (*Il-1B*, *IL-6*, *CCL4L2*, *CCL10*, *CCL4L2*) by senescent polyploidising cells, the sensing of cytosolic DNA produced by MS was at least partially protected from the innate and inflammatory immune response, particularly at the beginning of the MS process. 

#### 2.2.3. GO Enrichment Analysis of DE Bivalent Genes Reveals Reproductive Modules

Along with the powerful cytokine signaling and sensing of cytosolic DNA, we found the biological processes “multiorganism reproduction process” and ”reproductive structure development” (Figure 3, framed in white) on day 5 among the GO modules of upregulated bivalent genes. The former (GO:0044703) lists, among other processes, meiotic cell cycle and female pregnancy, and includes among other processes “ovarian nurse cell to oocyte transport” [14]. The definition of the “reproductive structure development” process reads: “The reproductive developmental process whose specific outcome is the progression of somatic structures that will be used in the process of creating new individuals…” (GO:0048608). The latter reproductive module and also “molting processes” (which in our context may correspond with excystation and budding from giant cells) were found on day 16; however, subsequently this process of “creating new individuals” progressed to “maternal placenta development” (Figure 4, all reproductive modules framed in white). The cells also show upregulation of locomotory behavior and migration compatible with their amoeboid phenotype, highlighted by the development of a powerful, microtubule-actin-rich cytoskeleton and budding offspring from late polyploidy giant cells, as described by us previously [4] and detailed further below.

The GO modules among the downregulated bivalent genes were not significantly enriched on days 5 and 16. Notably, on day 8, the downregulated bivalent genes show enrichment for the circadian rhythm GO BP (File S2), suggesting circadian deregulation removing cells from the normal cell cycle, which was associated in our previous work with senescence, MS, polyploidy, and a cancer soma-to-germ transition [9,15].

#### 2.2.4. The GO Enrichment of All DE Genes on Day 16

**Figure 5:**
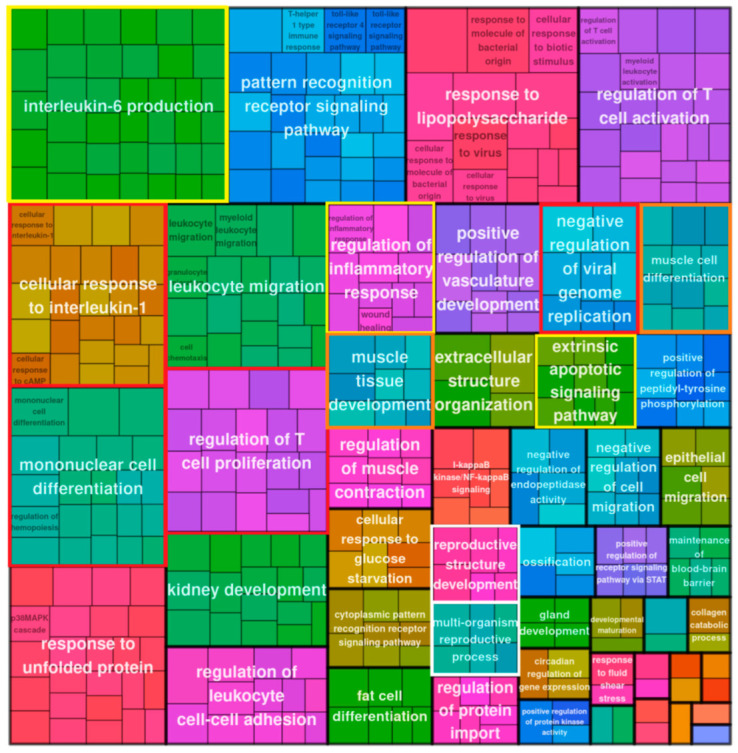
Treemap plot representation of the GO BP enrichment analysis results (GO modules with hypergeometric test pAdj < 0.05 classed as enriched) for all genes upregulated in MDA-MB-231 cells on day 16 post-DOX.

At this stage, the GO enrichment of all upregulated genes (Figure 5) revealed a considerable role of the cellular microenvironment and intercellular communication including the senescence secretome, hallmarked by the regulation of interleukin-6 and the cellular response to interleukin-1 (a master regulator of inflammation controlling a variety of innate immune processes), as well as the negative regulation of viral genome replication, the regulation of T-cell activation and mononuclear cell differentiation, the response to glucose starvation and the regulation of protein import, and the intrinsic apoptotic signalling pathway. In addition, the modules of “reproductive structure development” were also present (framed in white). As for the downregulated genes, the GO modules enriched among them mostly pertained to the mitotic cell cycle, and the regulation thereof (File S1).

Because of renewed interest in the atavistic theory of cancer [16], and an understanding of how cancer attractors during evolution are associated with polyploidy related to asexual reproduction, as recently identified in the TCGA database, including for breast carcinoma [17], we were interested in if and how these differences in gene expression changed the phylostratigraphic gene profiles.

#### 2.2.5. Phylostratigraphic Distribution of the Differentially Expressed Genes over Time after DOX-Treatment

**Figure 6:**
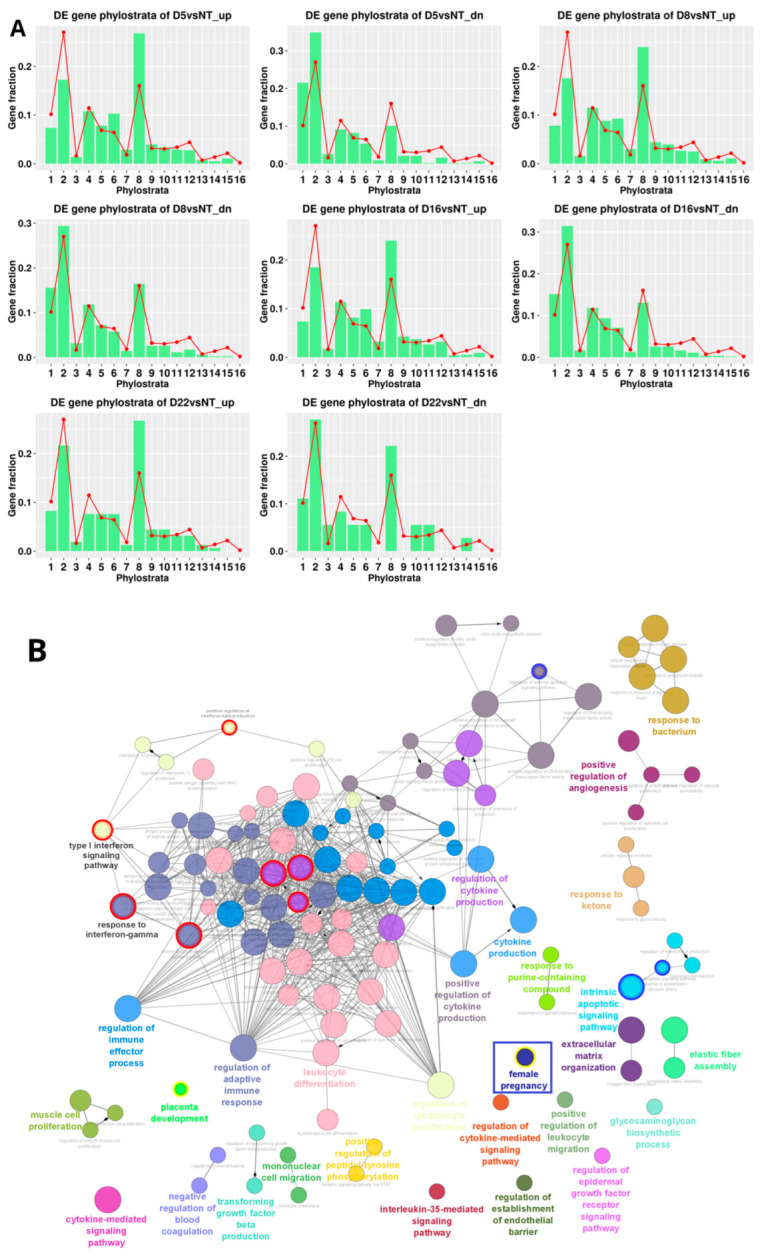

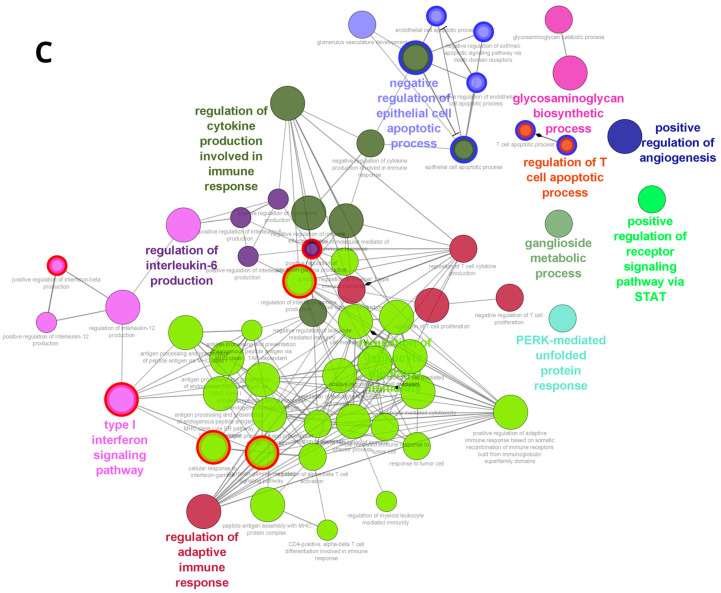
Phylostratigraphic analysis of MDA-MB-231 cells over the time course after DOX treatment. (**A**) The phylostratigraphic distributions of differentially expressed (up- and downregulated, abbreviated as “up” and “dn”) genes on days 5, 8, 16, and 22 post-DOX, with the whole-genome phylostratigraphic distribution (red line) serving as the background reference. (**B**) The ClueGO enrichment map representation of GO biological processes enriched (hypergeometric test pAdj < 0.05) in the STRING network of day 5 versus NT upregulated genes corresponding to the eighth phylostratum. The presence of both adaptive and innate immunity modules (including interferon-related pathways), angiogenesis, apoptosis, and female pregnancy (blue-framed) is noteworthy. (**C**) ClueGO enrichment map representation of GO biological processes enriched (hypergeometric test pAdj < 0.05) in the STRING network of day 16 versus NT upregulated genes corresponding to the eighth phylostratum. The modules of interferon-related pathways are circled in red; those pertaining to apoptosis are circled in blue. Figure 6B,C can also be viewed interactively in NDEX data base https://www.ndexbio.org/#/networkset/326907d3-a2f3-11ed-9a1f-005056ae23aa?accesskey=d13703d9d6c991087b20776c62648d36c27d4d92e7c4fea1b7e177a03221963b.

The results are presented in Figure 6A, with the general phylostratigraphy histogram of human genes used as a background. We see that the most crucial change occurred on day 5, with the relative downregulation of genes originating in Phylostratum 2, corresponding to unicellular Eukaryota (responsible for the evolving cell cycle, DNA damage signalling, and recombination repair mechanisms [15,18]) and the upregulation of the eighth multicellular phylostratum Euteleostomi. Phylostratum 6 (Bilateralia) associated, in particular, with polyploidy-cancer-linked angiogenesis [9] is also slightly upregulated. On days 8 and 16 the gene balance situation remained the same. On day 22, the suppressed phylostratum 2 partially reverted to the control (along with a return to the normal cell cycle).

In the upregulated eighth phylostratum of our DOX-treated material, the ClueGO enrichment map representation of GO biological processes enriched in the STRING network of day 5 versus NT upregulated genes revealed (Figure 6B) a dominant network of inflammatory cytokine production, which is consistent with a cellular senescence secretome and also immune response including a STING / Type 1 interferon component. Both senescence and immunity emerged around this period during evolution [18]. The module “response to bacterium” which reacts to soluble self-, mitochondrial, and viral/bacterial DNA as well, may be associated with cytosolic DNA accompanying mitotic slippage. In addition, a GO module designated as “female pregnancy’’ was revealed (Figure 6B, framed). The “female pregnancy” module (GO:0007565) is part of “multicellular organism development” and includes, mainly, the embryo implantation and maternal process involved in female pregnancy, in addition to multiple sub-sub networks, including secretion by tissues and viral processes. The latter paradoxically relates to placental biology [5], expanded further in the Discussion section. The upregulated genes of the module “female pregnancy” in our DOX-treated material include: FOS (logFC = 1.48) and JUNB (logFC = 2.40), indispensable regulators of embryonic and cancer stem cells with a positive loop to OCT4 [19]; IL-1β, a potent inflammatory cytokine involved in host defence through innate immunity (logFC = 5.58); VEGFA (vascular endothelial growth factor), acting in placenta and dysregulated in senescent and cancer cells (logFC = 1.82); THBD (thrombomodulin; logFC = 1.52); AREG, a ligand of the EGF receptor/EGFR, which regulates invasive phenotypes in cancers (logFC = 2.23); AGT (angiotensin; logFC = 3.38); PGF (placenta growth factor; logFC = 1.19); PTHLH, an intra/autocrine/paracrine parathyroid hormone-like-hormone found in the placenta DNA library [20] causing humoral hypercalcemia, and a poor prognosis marker in many cancers [21] (logFC = 2.06); and STC2 (stanniocalcin–hypocalcemic action) a universal tumor marker [22] (logFC = 2.21). In addition, the modules of the foetal-maternal interface such as the regulation and establishment of an endothelial barrier and the negative regulation of blood coagulation are also included.

Thus, GO modules for reproductive processes appeared in all in silico analyses. In Figure 6C, showing the network of phylostratum 8 on day 16, in addition to interleukin 6 and type 1 interferon signaling, the STAT pathway was revealed. The JAK/STAT pathway can be related to the trophoblast-like biology of the reproductive process [23] through the stress-activated MAPK cascade, which was also bivalently activated on day 16 (Figure 4). 

In summary, in all of our in silico transcriptome studies, the emergence of reproductive modules (soma-to-germ transition), co-opting with sensing and the response to cytosolic DNA linked through pervasive “female pregnancy” to the placental module, was found, along with the accumulation of super-giant cells by repeated MS. 

### 2.3. Immunofluorescence (IF) and Clonogenicity Studies

#### 2.3.1. Change of Cell Fate through MS on Days 4–5 Is Hallmarked by Soma-to-Maternal Germ Transition

**Figure 7:**
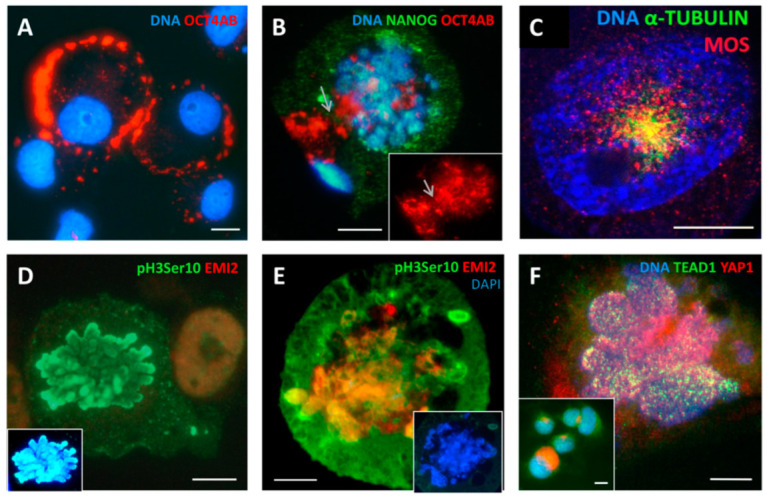
Cell fate change during MS where maternal germline specification and the inactivation of the Hippo pathway are observed on days 4–5 post DOX treatment: (**A**) OCT4-antibody-positive cells in NT control showing the cytoplasmic location of OCT4B; (**B**) the entrance of OCT4A into the cell nucleus (likely from the centrosome pole, arrowed) during MS; (**C**) the meiotic kinase MOS and α-TUBULIN form a monopolar spindle (arrowed) in the early prophase; (**D**) mitotic marker pH3ser10-positive and EMI2-negative metaphase in NT control; insert: metaphase-pH3ser10 with DAPI; (**E**) the chromosomes swelling and fusing in MS while losing the pH3Ser10 label become positive for EMI2 (insert: only DAPI staining); (**F**) the inactivation of the Hippo pathway, with the transition of YAP1 in its active form along with MS from the cytoplasm into the cell nucleus and interacting there with its partner transcription factor TEAD1 (insert: NT control cells). Imaged in RGB optical filters. Bars = 10 µm. Figure 7C is republished from [4] with an open access CC BY 4.0 license.

As indicated above, the reproductive process was highlighted by transcriptome studies from day 5 to day 16 after DOX treatment. Although previously we associated MS with meiotic traits based on IF and qPCR investigations [4], the mechanism of this transition remained unclear. Here, we noted the entry of OCT4A into the cell nucleus coinciding with MS (Figure 7A,B). To explore this further, fixed preparations were stained with an antibody for both A- and B-forms of OCT4. While in the NT sample, OCT4 was expressed in the cytoplasm (Figure 7A) corresponding to the spliced B-form lacking the first exon required for transactivation; after DOX treatment and the induction of MS, we found the clear transition of OCT4 into the reconstituting cell nuclei from the centrosome pole, on days 4–5 (Figure 7B). The shift between full A- and spliced B-form of the *POU5F1* gene can occur by the reversible methylation of its enhancers [24]. In our previous work using qPCR and digital PCR, we observed a two-fold increase in *OCT4A* transcription when referred per gene on day 4 (republished in Figure 1C,D). The importance of this observation is underscored by the role of *OCT4A (POU5F1)* as a maternally expressed germline-specifying factor [25,26]. The link of mitotic slippage to maternal soma-to-germ transition is also in line with the previously reported activation of the meiotic kinase MOS (Figure 7C) and the expression of several meiosis-specific genes found by qPCR and IF on days 4–8 (Figure 1C). Here, we also found the upregulation of EMI2, the co-activator of MOS, in the oocyte meiotic arrest of the anaphase-promoting complex during MS (Figure 7D,E). 

In addition to these hallmarks of soma-to germ transition associated with mitotic arrest and slippage, we also decided to study the YAP1/TEAD1 distribution as a possible link between the sensing of cytosolic DNA in MS and the inactivation of the Hippo pathway as reported in the literature [27]. Here, we confirmed on day 5–8 post DOX treatment this important hallmark of cell-fate change—i.e., the inactivation of the Hippo pathway, with the transition of YAP1 along with MS from the cytoplasm into the cell nucleus and interacting there with its partner transcription factor TEAD1 (Figure 7F). The reproductive modules of “female pregnancy” and “maternal placenta development”, as well as the upregulated genes, indicate the presence of placenta components seen during evolution. Therefore, we suspected that polyploid giant cells may recapitulate a function of the atavistic intercellular communication placed in placenta development, precursors that further evolved in mammals as embryo invasion and the ”fetus-mother” trophoblast relationship. In this context, we decided to investigate the cellular location and functionality of CDC42-kinase, a small Rho GTP-ase which is engaged in trophectoderm lineage specification in mammals [28].

#### 2.3.2. Inhibition of the Trophectoderm Lineage Specifier CDC42 Suppresses the Clonogenic Survival of DOX-Treated MDA-MB-231 Cells

CDC42, together with the substrate-phosphorylating RAC1 component, is indispensable in the biology of various solitary and social amoebae, parasitic protists, budding yeast, and mammalian animals, including female pregnancy. It was also shown to promote tumor progression and metastases, particularly in triple-negative breast cancer [29]. The activated CDC42 acts by modulating the structure of actin and tubulin dynamics, creating and modifying the cytoskeleton, cell–cell interaction, and multiple invasion processes [30]. In amoeba, CDC42 participates in the excysting of spores from macrocysts, the same function as for budding yeast. In humans, CDC42 is also involved in the creation of the immunological synapse [31]. In relation to female pregnancy, activated CDC42 has two membrane invasive functions, polar body emission and placentation, with the migration and invasion of the human extravillous trophoblast, where CDC42 is directly located in microvilli [32,33]. In our previous work, we highlighted the initial increase in CDC42 transcription on days 4–5 after DOX treatment and the many-fold accumulation of transcripts in polyploidy giant cells, due to high gene dosage (presented here in Figure 1D).

**Figure 8:**
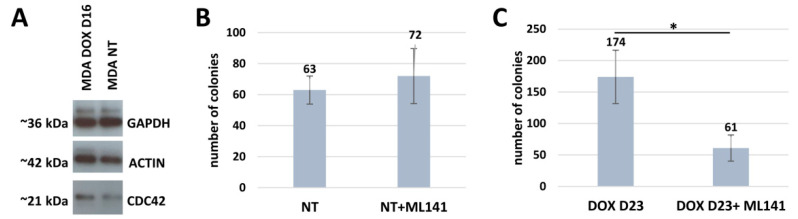
The evaluation of CDC42 by WB and clonogenicity assay: (**A**) Western blot analysis of CDC42 expression in MDA-MB-231 cells before and after DOX treatment. The GAPDH antibody is used as a loading control. (**B**,**C**) The evaluation of clonogenicity after DOX treatment and CDC42 suppression. (**B**) Clonogenicity of MDA-MB-231 NT cells on day 23 after CDC42 inhibitor, ML141, treatment (100 cells seeded per well). (**C**) Three-fold decrease in the clonogenicity of MDA-MB-231 cells after DOX and CDC42 inhibitor, ML141, treatment (counted from ~1.5 × 10^6^ cells initially seeded per flask) on day 23 after treatment evaluated in five independent experiments. * *p* < 0.05.

The presence of CDC42 in NT and DOX-treated cells was confirmed by Western blotting (WB) (Figure 8A). Using the inhibitor of CDC42, ML141, we found a three-fold suppression of the clonogenicity of the DOX-treated MDA-MB-231 cells, in five independent experiments scored on day 23, although the colony formation capability on NT control cells was not significantly changed (Figure 8B,C). The stained colonies are shown in Supplementary Figure S1.

#### 2.3.3. CDC42 Is Located at the Periphery, Buds, and Their Microvilli of Late DOX-Treated Polyploid Giant Cells

**Figure 9:**
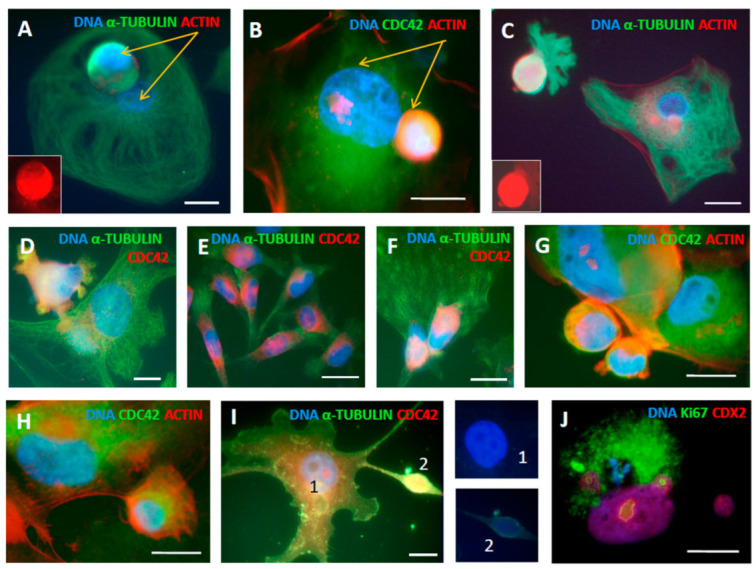
CDC42 kinase acting together with actin and tubulin in late (second–third week) DOX-treated polyploid giant MDA-MB-231 cells participates in collective activities for survival: budding, homing, invasion, and DNA transfer: (**A**–**D**) giant amoeboid cells on days 13–18 post-DOX treatment budding mobile spore-like sub-cells, which are highly enriched in actin and tubulin and contain CDC42, particularly in the microvilli (in **D**); (**A**,**B**) arrows indicate two type of subnuclei, one in the mobile bud and another immobile remaining at place; (**E**) CDC42 is found in the cytoplasm of NT control cells; (**F**) CDC42-rich small sub-cells located on the surface of the polyploid giant cell; (**G**) CDC42 is found intensely at the periphery of the polyploid giant cell possibly providing homing for their smaller neighbors; (**H**) a giant cell budding a sub-cell; (**I**) the participation of CDC42 in branching the structures filled with diffuse DNA; (**J**) the trophectoderm lineage marker CDX2 weakly positive in a polyploid giant cell nucleus and its two buds, on day 19 after DOX treatment. Bars = 20 µm. Figure 9A,C are reproduced from [4] with an open access CC BY 4.0 license.

The immunofluorescent detection of CDC42 in the chamber slide cultures, showed CDC42 to be highly enriched in the polyploid giant cell buds (appearing at the end of the second week post-DOX and beyond), which are also very rich in actin and tubulin and occasionally can be found asymmetrically originating beside a sub-nucleus lacking these components (Figure 9A–D, E for NT control). When outside the polyploid giant cells, these buds often display the peripheral microvilli enriched with all three components: actin, tubulin, and CDC42, indicating their mobility and invasive capacity (Figure 9C,D).

In some cases, such small cells were seen branched from the polyploidy giant cells on a thin actin “foot” (Figure 9H). In addition, CDC42 staining was more intense at the periphery of these cells where their smaller CDC42-enriched offspring/neighbors may be homing (Figure 9G). The participation of CDC42 in ejecting the structures filled with diffuse low-density DNA was also seen (Figure 9I). The staining for the preimplantation trophectoderm lineage specifier, CDX2, in late giant cells showed weak co-staining with Ki67 in polyploid giant cells and their intracellular buds, with accumulation in the autophagosomes but absence in the recovered cell nuclei (Figure 9J). 

It is somewhat difficult to ascertain when the mobile CDC42/ACTIN/TUBULIN-enriched buds are ejected from giant cells and when they are homing to them. In the current study, we tried to clarify how the budding cancer “babies” develop from the “maternal germ cell” and the remnant super-giant with the features of the “maternal placenta”, as extrapolated from our in silico analysis, to attain different fates. 

#### 2.3.4. DNA Repair, Autophagy, and DNA Damage Sorting in Small and Super-Giant Cells

**Figure 10:**
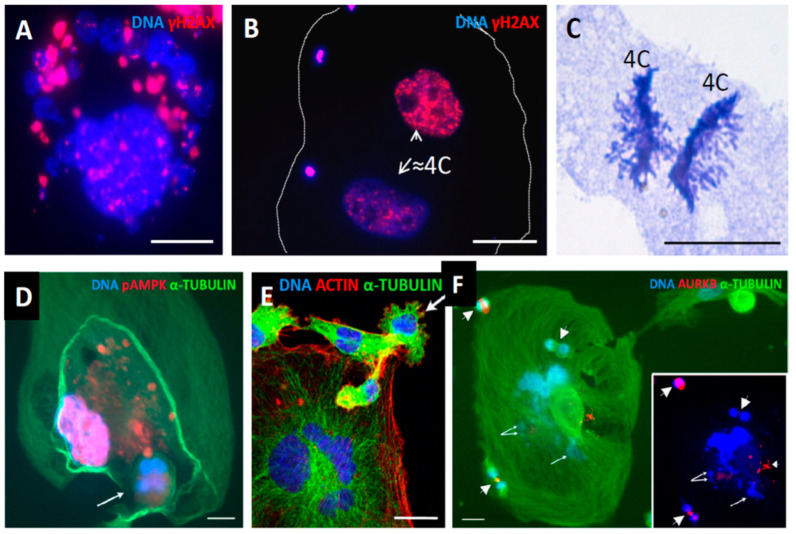
The production of fragmented cytosolic DNA in repeated cycles of MS in polyploid giant cells; its separation from the repairing sub-nucleus by autophagy and support from a polyploid giant for the budding and homing of mitotic survivors. (**A**) MS positive for DNA DSBs (second week post DOX). (**B**) A giant cell with two ~4C sub-nuclei, one is γH2AX positive, while the other is free of DNA DSBs, and three small clusters of the sorted DSB-enriched DNA at the cytoplasm periphery. (**C**) Bi-polar anaphase of the equal 4C binemic chromosome groups from an 8C MDA-MB-231 cell–a clear indication of reduction division—on day 19 post-DOX-treatment. (**D**) The possible excystation of a tetra-nuclear subcell (arrow) leaving a giant cell, while another subnucleus (~8C) is positive for pAMPK, indicating its autophagic degradation (republished from [4]). (**E**) A giant multinuclear cell is budding a sub-cell (arrow) from a fragment of a polyploid giant cell 7 weeks post-DOX treatment, expelling a daughter cell, with the actin twisting around the anaphase spindle. (**F**) A super-giant amoeboid cell with a deteriorating nucleus (arrowed, better seen on the DAPI-stained image in insert) homing three small cells performing mitosis (arrowheads): metaphase, anaphase, and telophase. Bars = 25 µm. (**E**,**F**) republished from [4] with an open access CC BY 4.0 license; (**C**,**D**) from [11], the article can be found https://www.researchgate.net/publication/322581015.

The asymmetric a-mitotic segregation of polyploid giant cancer cell subnuclei in relation to DNA repair was already reported by us in this [4] and other models of genotoxic treatment. The whirling of the whole polyploid giant cell genome and the rotation at the nuclear periphery, together with the looping nuclear lamin, presumably in search of homology for the recombination repair and sorting of the damage by autophagy, has been proposed and partially examined [34,35,36,37] (occurring at the brink between survival and death by mitotic catastrophe, indirectly supported in studies of anastasis (return from apoptosis)) [38,39]. Therefore here, in this model, we present just a few examples (Figure 10A,B,D) of DNA damage sorting with the participation of autophagy (pAMPK-positivity in Figure 10D) and two examples of reduction division (Figure 10C,E), as well a unique picture of a polyploid super-giant cell homing three offspring just at the state of mitosis, while its own nucleus is deteriorating (Figure 10F). We also know from dozens of experimental repeats that the super-giants die alongside the establishment of the mitotic clonogenic growth, as exemplified in Figure 1A [4]. 

**Figure 11:**
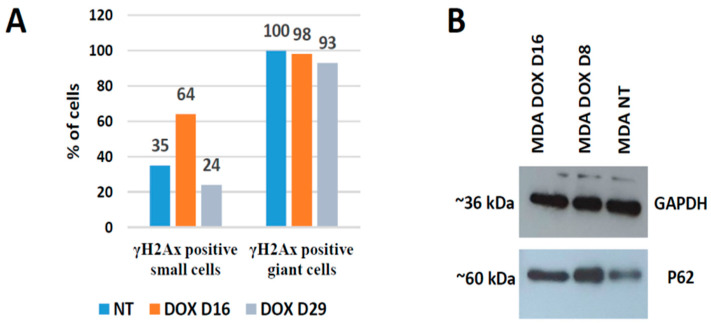
DNA damage, repair, and autophagy in MDA-MB-231 cells after DOX treatment. (**A**) Microscopic counts of gamma-H2AX-positive cells showing recovery after DOX of small cells without DNA DSBs, while late polyploidy giant cells remain with DNA breaks. (**B**) Western blot analysis of autophagy marker p62 expression in NT control cells and after DOX treatment. The GAPDH antibody is used as a loading control at the same time points.

Accordingly, it appears that polyploid giant cells sort the DNA damage between subnuclei and deliver small viable survivors, but themselves only produce even more cytosolic DNA, degraded by autophagy for recycling, and also energy to feed the offspring and to stimulate their mitoses, also by a secreted microenvironment, until they are self-sufficient and capable of forming clones. Additional data are presented in Figure 11.

It is seen that the polyploid giant cells (large nuclei) remain after DOX treatment predominantly with DNA DSBs, while the small cells (presumably delivered from them) become repaired. WB analysis of the DNA autophagy mediator p62 (Figure 11B), shows its upregulation in terms of DNA-damage response, in line with literature [40].

## 3. Discussion

We began our observations of the basal breast cancer cell-line MDA-MB-231′s response to DOX by finding the replication stress and under-replication of DNA in the late S-phase. This response has several consequences. First, as shown by us previously, it produces DNA damage, telomere attrition, and cytosolic DNA, and induces accelerated senescence [4]. The DNA under-replication changes cell fate by DNA-damage signaling and by preceding replicative stress [41].

To be precise, the replicative stress and under-replication induce a soma-meiotic germ transition, leading to metaphase arrest and slippage (most likely in the second altered cycle preceded by aberrant mitosis). MS is mediated by cooperation between the meiotic kinase MOS, which suppresses the degradation of cyclin B, and EMI2 (both preventing the induction of the anaphase-promoting complex). MS apparently belongs not to a mitotic but rather a female meiotic cell cycle, leading to a state similar to oocyte maturation, in expectation of fertilisation or parthenogenetic stimulus. Both stimuli should start embryonic development by cleavage divisions of blastomeres [42,43,44], which are considered by Niu and colleagues as equivalent to polyploid giant cancer cells [45]. 

In line with our earlier observations, this soma-germ transition state appears in a transcriptome analysis as the earliest feature of reproduction on the same 4–5 days with the GO module “multiorganism reproductive process”. Secondly, in parallel with MS, the pro-inflammatory immune response is activated, sensing the cytosolic DNA through the cGAS-STING pathway [46]. This soluble, unrepaired, and cleaved-by-autophagy DNA accumulates along with duplications of the DNA in the polyploid cells with each subsequent MS cycle. It is important to note that the soma-germ transition induced by MS also coincided with inactivation of the Hippo tumor-suppressor pathway and the transition of YAP1 in the cell nucleus, colocalising and activating TEAD1, along with the nuclear activation of OCT4A. Accordingly, the meiotic soma-germ transition of maternal origin resulting in MS was accompanied by YAP1/Hippo nuclear-cytoplasmic redistribution, known as sensing cytosolic DNA, resulting from the same MS as referred to in [27], and likely circularly linking both events. In turn, and thirdly, the accumulating soluble DNA induces the innate and adaptive immune response (transcriptionally, although requiring evidence in vivo) increasingly revealed by the activation of relevant genes in the PPI STRING network in the super-giant cancer cells. In its turn, and fourthly, this soluble DNA was not only used for re-utilisation but was also related to the induction of the maternal placenta module from day 5 onwards, as seen in the GO biological process of “female pregnancy”. The latter was best revealed by bivalent genes and particularly in the DOX-upregulated eighth gene phylostratum (integrating the appearance of cellular senescence and immunity in multicellular organisms through evolution [18]). 

Therefore it is interesting that recently we revealed oocyte maturation and meiotic cell division in the STRING PPI network of the TCGA database BRCA (and some other cancers) patient sample cohort in association with whole genome duplications [17]. So, the current in silico and immunofluorescence data in our MDA-MB-231-DOX model confirm the reality of such cell-fate change of somatic cancer cells. Notably, this pathway takes place not only in female but likely also triploid male cancers, through the doubling of the maternal genome [47]. 

However, the question remains, how does the mature maternal cancer “germ” originating from somatic cells establish a link to “maternal placenta development”? Here, we should recall the viral component. In our transcriptome analysis, the response to viral cell cycle and bacterial molecules, both positive and negative, evolutionarily emerged in multicellular eukaryotic cells for defense from viral and bacterial infection as the c-GAS-STING-interferon-related immunity pathway was found. During MS, this pathway is activated presumably through the fragmentation of the self-DNA. Concurrently, the under-replication of heterochromatin would also cause its de-methylation and the activation of retroviral DNAs concealed there, which ultimately can end in cell death by transposition [48]. The situation is potentially even more complex, because Edward Chuong [5] showed the domestication of endogenous retroviruses (HERV) in the human genome, enabling, in his opinion, the evolution of proto-mammals. 

These endoviruses, which constitute 8% of the human genome, can produce syncytin, causing cell–cell fusion that enables the creation of the syncytiotrophoblast for the separation of the fetal and maternal bloodstreams, preventing mutual destruction by competing immune systems. In this way HERVs are at work in the human placenta. Moreover, the activated HERVs were found by Diaz-Carballo and colleagues [49] to be mobilised in drug-treated polyploid giant cancer cells, participating in horizontal gene transfer together with mitochondrial DNA through ejections, similar to that described here in Figure 9I (and also observed by us in patient material; unpublished observations). Moreover, the same group described the mitochondrial encapsulation around cell nuclei of giant cancer cells resulting from genotoxic treatments [49], while Aarreberg et al. [50] reported that interleukin-1β induces a mitochondrial DNA release to activate innate immune signaling via cGAS-STING. Moreover, interleukin-1β from the phylostratum 8, upregulated by DOX, as revealed here 48-fold, is a component of the GO “female pregnancy”. Collectively, this data potentially provides a unifying resolution for the various aspects of the response: MS, resulting from cell senescence and energised by active mitochondria (counteracting cell death by anastasis [39]), both releases fragmented self-DNA and activates HERVs. Through the inflammatory interleukin-1β immune response and cGAS-STING signaling, these programs become linked with the induced trophectoderm and placental proto-genes. 

Returning to the female pregnancy module, it presumes not only the ectopic expression of placental genes, but also the presence of an “embryo proper” to invade. The MS preceded by replication stress supports the conversion of cancer somatic cells into a mimic of the arrested “mature oocyte” awaiting fertilisation or parthenogenetic stimulus to start early embryo development. Building on this observation, we propose a hypothesis that one of the known carcinogenic placental hormones, PTPLH, when activated, can intra-autocrinally and paracrinally elevate the humoral calcium level. *PTPLH* was isolated from a placental DNA library by Yasuda et al. [20], it regulates fetal-placental calcium transport [51], and is a poor prognosis marker in several somatic cancers [21]. PTPLH activity may also counteract calcium precipitation in the acidic microenvironment typical of cancers [52]. Calcium elevation mimics fertilisation and is widely used to cause parthenogenesis in experiments and domestic animals [53]. In this way, chemotherapeutic drugs can induce MS and therein, in a stressed senescent cancer cell, two key components—the imitation of a mature egg arrested before fertilisation and a placental imitation, which can cause the parthenogenesis of this arrested “egg” (formation of the embryo proper) in the same super-giant multinucleated polyploid cell. The latter, in addition, also serves to feed and home the delivered offspring. In other words, a system of “female pregnancy” is obtained, regulated by positive feedback, including the secreted microenvironment, which creates a self-reproducing metastatic cancer. 

Innate immunity has a very important role in normal pregnancy [54], with the strictly controlled processes allowing the baby to be nourished whilst the mutual immune destruction of both baby and mother is prevented. In view of the obtained results, the evasion mechanisms of tumor immunity may be related to the evolutionary fetal-maternal relationship.

Cdc42, an invasive placental component, shown here as intrinsically involved in the activities of the super-giant polyploid cancer cells, developed post-MS and favored the resistant clonogenic survival of DOX-treated MDA MB 231 cells. As a result, the targeting of CDC42 may be an attractive therapeutic strategy for preventing drug resistance. Strategies to target CDC42 are already available [29,55]. Similarly, other therapeutic strategies are also worthy of consideration. For example, the use of anti-viral medicines for treating cancers more generally, as well those aimed directly against HERV, have been undertaken [56]. Bisphosphonates, decreasing lipid droplets to target senescent chemo-resistant polyploid giant cancer cells, were also recently described [57]. Potentially, this treatment with zolendronic acid can decrease humoral calcium and interrupt the parthenogenetic link of the “female pregnancy system” revealed here in polyploid giant cancer cells. These approaches likely deserve attention and development in the future. 

## 4. Materials and Methods

### 4.1. Cell Line and Treatment

The breast adenocarcinoma MDA-MB-231 cell line (triple-negative, modal chromosome number 64) was obtained from the European Collection of Authentic Cell Cultures (ECACC, Wiltshire, UK). The cells were grown in flasks or on chamber slides in Dulbecco’s Modified Eagle’s Medium (DMEM) supplemented with 10% fetal bovine serum (FBS; Sigma-Aldrich, St. Louis, MO, USA) at 37 °C in a 5% CO_2_ humidified incubator without antibiotics. For the experimental studies, the cells were maintained in the log phase of growth and treated with 100 nM DOX (doxorubicin) for 24 h. After drug removal, the cells were maintained by replenishing the culture medium every 2–3 days and sampled over a 3-week period post-treatment until the appearance of escape clones. To suppress CDC42 activity cells after DOX removal, the cells were treated with 20 μM of the CDC42 inhibitor, ML141 (Sigma-Aldrich, St. Louis, MO, USA) until the appearance of escape clones. The concentration of ML141 as CDC42-inhibitory in the MDA MB 231 cells was based on [55].

### 4.2. Cell Colony Formation

Preliminary experiments showed that colony formation after DOX treatment was very low; therefore, to determine the colony formation ability after DOX treatment, it was performed in T25 flasks (along with immunofluorescence experiments carried out from the parallel T25 flasks). In a T25 flask ~ 1.5 × 10^6^ cells were seeded and treated with 100 nM DOX, alone, or in combination with 20 μM ML141. For NT control, cells were also seeded in 6 well plates (100 cells per well). On day 23 after treatment, the cells were rinsed with phosphate buffered saline (PBS), then fixed in methanol for 10 min and stained with 0.5% crystal violet solution (in 25% methanol) for 10 min, rinsed with ddH2O, air dried, and the number of the eye-visible colonies counted. The colony formation capability was calculated from the initially seeded 1.5 × 10^6^ cells per T25 flask on day 23 after treatment with DOX and ML141. For the NT control, the clonogenicity was calculated on day 23 after ML141 treatment from initially 100 cells seeded per well. The Student’s *t*-test for unpaired samples was used to calculate the statistical significance of the difference of means (GraphPad Software Inc.). Statistical significance was accepted with *p* < 0.05.

### 4.3. Immunofluorescence (IF)

Standard IF staining was performed according to the procedures detailed previously [4]. Briefly, the cell cytospins or chamber slides were fixed in methanol for 7 min at −20 °C, dipped 10 times in ice-cold acetone, and allowed to briefly dry. When staining for α-tubulin and actin, the post-fixation drying step was omitted and fixation in 4% paraformaldehyde with a triple wash in PBS was performed. Blocking for 15 min in Tris buffered saline (TBS), 0.05% Tween 20%, and 1% bovine serum albumin (BSA) at room temperature followed. Incubations with primary antibodies overnight at 4 °C and appropriate secondary antibodies (goat anti-mouse IgG Alexa Fluor 488, goat anti-rabbit IgG Alexa Fluor 594 (Invitrogen, Carlsbad, CA, USA)) for 40 min at room temperature were carried out. The samples were counterstained with DAPI (0.25 μg/mL) and embedded in Prolong Gold (Invitrogen, Carlsbad, CA, USA). The primary antibodies and their sources are listed in Table 2.

For microscopic observations, a fluorescence light microscope (Leitz Ergolux L03-10, Leica, Wetzlar, Germany) equipped with a color video camera (Sony DXC 390P, Sony, Tokyo, Japan) and laser scanning confocal microscope (LEICA TCS SP8, Wetzlar, Germany) were used.

### 4.4. Toluidine Blue DNA Staining and Image Cytometry

Toluidine blue DNA staining and image cytometry were performed as detailed previously [4]. The cytospins were fixed in ethanol: acetone (1:1) for ≥30 min at 4 °C, air-dried and hydrolyzed with 5 N HCl for 20 min at room temperature. The slides were then washed in distilled water (5 × 1 min), stained for 10 min with 0.05% toluidine blue in 50% citrate-phosphate McIlvain buffer at pH 4, and rinsed with distilled water. The samples were then blotted dry and incubated twice in butanol for 3 min each at 37 °C and twice in xylene for 3 min each at room temperature before being embedded in DPX. The DNA content was measured as the integral optical density (IOD), using Image-Pro Plus 4.1 software (Media Cybernetics, Rockville, MD, USA). The stoichiometry of DNA staining was verified using the values obtained for metaphases compared with anaphases and telophases (ratio 2.0); the (2C) DNA values in arbitrary units were averaged from measuring the anaphases in untreated tumor cells. For the cell cycle measurements, 200–500 interphase cells were collected at each point.

### 4.5. RT-PCR and Selfie-Digital RT-PCR

The total RNA was extracted from MDA-MB-231 (10^6^) cells using TRIZOL (Invitrogen, Carlsbad, CA, USA) and for the Selfie-digital RT-PCR the cells were lysed in the 100ST DNA/RNA/protein solubilization reagent (#DCQ100ST, DireCtQuant, Lleida, Spain) at 250,000 cells/mL. RT-PCR and Selfie-digital RT-PCR was performed and the primers were used as described in [4].

### 4.6. Western Blot Analysis

Living adherent cells were washed with PBS, harvested into an ice-cold RIPA lysis buffer using a cold plastic cell scraper, and centrifuged at 10,000× *g* for 20 min at 4 °C. The concentration of proteins was estimated with the Qubit protein Assay kit (Invitrogen, Carlsbad, CA, USA). Equal protein loading in each lane was checked by Ponceau S staining. SDS PAGE was used to separate 20 μg of each protein sample on 15% gels and then blotted onto nitrocellulose membranes (Amersham, Buckinghamshire, UK). The membranes were blocked in 3% BSA dissolved in TBS containing 0.1% Tween-20 for 1 h at room temperature (RT). Subsequently, the membranes were probed overnight at 4 °C with the primary antibodies listed in Table 2. The respective proteins were detected after incubation with horseradish peroxidase-conjugated secondary antibodies (1:2000, rabbit anti-mouse IgG-HRP, 61-6520, Invitrogen; goat anti-rabbit IgG-HRP 32460, Thermo Fisher Scientific, Rockford, IL, USA), using an ECL system (Thermo Scientific, Rockford, IL, USA) according to the manufacturer’s instructions.

### 4.7. Transcriptome Library Preparation

RNA isolation was performed using the AllPrep DNA/RNA/miRNA Universal Kit (Qiagen, Hilden, Germany). RNA quality was determined using the RNA 6000 Pico Kit and Agilent 2100 bioanalyzer (Agilent Technologies, Santa Clara, CA, USA). The cells for RNA isolation were collected on days 0, 5, 8, 16, and 22. The experiments were performed in triplicate. A transcriptome library preparation was performed using the MGIEasy RNA Directional Library Prep Kit (MGI, Shenzhen, China). The Veriti 96 Well Thermal Cycler (Applied Biosystems, Bedford, MA, USA) was used to carry out all reactions and incubations as intended in the library preparation protocol. The RNA enrichment was performed by depleting rRNA with the MGIEasy rRNA Depletion Kit (MGI, China). Work continued accordingly with the instructions for the 250bp insert size. The DNBSEQ-G400 sequencing platform (MGI, China) was used for sequencing the libraries. The RNA concentrations after extraction, dsDNA concentrations after amplification, and ssDNA concentrations after multiplexing were determined using the appropriate Qubit assays: Qubit™ RNA HS Assay Kit, Qubit™ dsDNA HS Assay, Qubit™ ssDNA HS Assay Kit, and Qubit^®^ 2.0 Fluorometer (Thermo Fisher Scientific, Rockford, IL, USA). The fragment size after amplification was determined using a high sensitivity DNA kit and Agilent 2100 bioanalyzer (Agilent Technologies, USA). The transcriptome FASTQ files were quality checked with a FastQC, trimmed of 3′ adapters and low-quality reads with Cutadapt (with parameters of -m 70 and -q 10), pseudo-aligned to the GRCh38.p13 human transcriptome (downloaded from the GENCODE site) with Salmon [58], and then the tximport package [59] in R was used to acquire gene-level count matrices for each sample.

### 4.8. Differentially Expressed Gene (DEG) Identification and GO Enrichment Analysis

A differential expression analysis comparing treated samples with the NT control at the D5, D8, D16, and D22 time points was performed using edgeR [60], obtaining the differentially expressed genes (DEGs) with the glmQLFTest approach. The threshold for differential expression was selected to be FDR < 0.05 and LogFC > 1 (in absolute value). The EdgeR’s plotMDS visualization function was used to construct a MDS (multidimensional scaling) plot of the samples, while volcano plots of the DEGs were generated with EnhancedVolcano [61]. The resulting lists of upregulated and downregulated genes for each time point were subsequently subjected to a Gene Ontology (GO) [14] enrichment analysis with the hypergeometric test method and Benjamini-Hochberg *p*-value correction (pAdj < 0.05 threshold for enrichment) implemented in the clusterProfileR [62] package. The enrichment results were visualized in treemap plot form using the rrvgo [63] R package.

### 4.9. Bivalent Gene Enrichment Analysis, Phylostratigraphic Analysis, and Eighth Phylostratum STRING Protein–Protein Interaction Network Analysis

The list of 3590 bivalent genes was obtained from Court and Arnaud, 2017 [64]. The DEG lists (upregulated and downregulated genes, separately) for each time point were assessed for statistically significant enrichment with bivalent genes using the binomial test method. The differentially expressed bivalent genes were assessed separately with a GO enrichment analysis and the results visualized as described in the previous section.

The whole-genome phylostratigraphy data separating the genes into evolutionary groups referred to as phylostrata was obtained from Trigos et al., 2017 [18]. The phylostratigraphic distributions of the differentially expressed genes at each of the time points were plotted with ggplot2 [65], with the whole-genome phylostratigraphic distribution serving as a reference.

Due to the prevalence of eighth phylostratum genes among the upregulated genes, it was further decided to subject these eighth phylostratum DEGs to a STRING PPI (protein–protein interaction) network analysis in order to determine the functional relationship between them. Protein–protein interaction data for these genes of interest was downloaded from the STRING database and visualized in network form using Cytoscape [66]. After extracting the giant component of each network, the resulting networks were subjected to a GO-enrichment analysis with the ClueGO [67] Cytoscape app. The resulting enriched GO modules were subsequently visualized in an enrichment map format.

## Figures and Tables

**Table 1:** The expression of differentially expressed (LogFC, compared to NT) genes sensing cytosolic DNA triggered by the AIM2 inflammasome.

Genes	Up/Down	D5	D8	D16	D22
*IL1B*	Up	5.58	4.78	3.87	NA
*IL6*	Up	3.77	3.23	2.73	NA
*CCL4L2*	Up	2.89	3.46	6.21	4.87
*CCL5*	Up	2.05	1.76	2.95	1.7
*NFKB1A*	Up	1.88	1.85	1.64	NA
*NFKBκB*	Up	NA	NA	1.26	NA
*CXCL10*	Up	1.86	1.44	NA	NA
*CASP1*	Up	1.38	NA	NA	NA
*RNF125*	Up	1.29	NA	1.06	NA
*AIM2*	Up	1.14	NA	NA	NA
*RELA*	Up	1.04	NA	NA	NA
*DDX58*	Up	NA	1.5	1.59	NA
*POLR3G*	Down	−1.25	−2.07	−1.47	NA
*POLR3K*	Down	−1.14	NA	−1.02	NA
*POLR2F*	Down	−1.16	−1.11	−1.09	NA
*POLR2L*	Down	−1.09	−1.11	−1.11	NA

**Table 2:** Antibodies used, their specificity, and source.

Antibody Against	Description	Specificity/Immunogen	Concentration Used	Product No. and Manufacturer
AURORA B	Rabbit polyclonal	A peptide derived from within residues 1–100 of human Aurora B.	1:300	ab2254, Abcam, Cambridge, UK
⍺-Tubulin	Mouse monoclonal	Recognizes an epitope located at the C-terminal end of the ⍺-tubulin isoform in a variety of organisms.	1:1000	T5168, Sigma-Aldrich, St. Louis, MO, USA
β-Actin	Mouse monoclonal	Synthetic peptide corresponding to human β-actin aa 1–100.	1:500 1:2000 WB	ab8226, Abcam, Cambridge, UK
CDC42	Rabbit polyclonal	The details of the immunogen for this antibody are not available.	1:100 1:500 WB	ab187643, Abcam, Cambridge, UK
CDC42	Mouse monoclonal	Specific for an epitope mapping between amino acids 166–182 at the C-terminus of CDC42 of human origin.	1:50	sc-8401, Santa Cruz, Dallas, TX, USA
CDX2	Rabbit monoclonal	A synthetic peptide corresponding to residues near the N-terminus of human CDX2.	1:50	MA5-14494, Thermo Fisher Scientific, Rockford, IL, USA
EMI2	Rabbit polyclonal	Recombinant protein corresponding to human EMI2.	1:100	PA5-55042, Invitrogen, Carlsbad, CA, USA
F-ACTIN		Phalloidin-iFlour 594 conjugate.	1:500	ab176757, Abcam, Cambridge, UK
GAPDH	Mouse monoclonal	Raised against recombinant GAPDH of human origin.	1:5000 WB	sc-47724, Santa Cruz, Dallas, TX, USA
γ-H2AX	Rabbit polyclonal	Recognizes human and mouse γ-H2AX.	1:200	4411-PC-100, Trevigen, Gaithersburg, MD, USA
MOS (C237)	Rabbit polyclonal	Epitope mapping at the C-terminus.	1:50	sc-86, Santa Cruz, Dallas, TX, USA
NANOG	Mouse monoclonal	Recombinant human Nanog.	1:50	N3038, Sigma-Aldrich, St. Louis, MO, USA
OCT4	Rabbit polyclonal	A peptide derived from within residues 300 to the C-terminus of human Oct4.	1:200	ab19857, Abcam, Cambridge, UK
p-AMPKα1/2 (Thr183/172)	Rabbit polyclonal	Epitope corresponding to phosphorylated Thr172 of AMPKα1 of human origin.	1:50	sc-101630, Santa Cruz, Dallas, TX, USA
pH3Ser10	Mouse monoclonal	Recognizes phospho- S10 on histone H3.	1:200	ab14955, Abcam, Cambridge, UK
P62/SQSTM1	Rabbit polyclonal	A synthetic peptide corresponding to human SQSTM1/ p62 (C-terminal).	1:500 WB	ab91526, Abcam, Cambridge, UK
TEAD1	Mouse monoclonal	Carrier-protein-conjugated synthetic peptide encompassing a sequence within the centre region of human TEAD1.	1:100	GT13112, Invitrogen, Carlsbad, CA, USA
YAP1	Rabbit polyclonal	Recombinant YAP1 protein expressed in bacteria.	1:400	PA-46189, Invitrogen, Carlsbad, CA, USA

## Supplementary Materials

The following supporting information can be downloaded at: https://www.mdpi.com/article/10.3390/ijms24043237/s1.
