# Supplementary figures and images for: The Role of Mitotic Slippage in Creating a “Female Pregnancy-like System” in a Single Polyploid Giant Cancer Cell
Source: Int J Mol Sci. 2023 Feb 6;24(4):3237. doi: 10.3390/ijms24043237 (PMC9960874; doi:10.3390/ijms24043237)

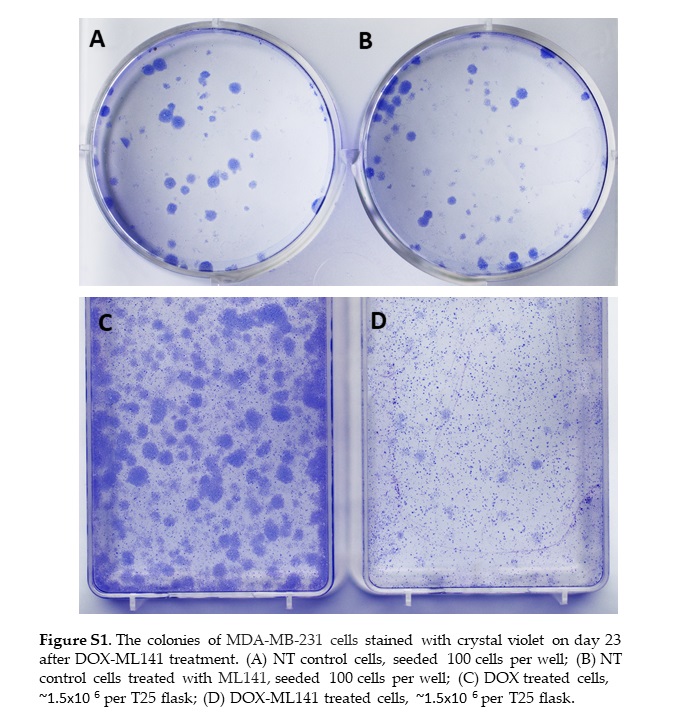

Supplement: Supplementary file 1 [file ijms-24-03237-s001.zip › Figure S1.jpg]
